# Immediate effect of trunk flexion and extension isometric exercise using an external compression device on electromyography of the hip extensor and trunk range of motion of healthy subjects

**DOI:** 10.1186/s13102-022-00506-1

**Published:** 2022-06-21

**Authors:** Tian-zong Huang, Suhn-yeop Kim

**Affiliations:** 1 Department of Physical Therapy, Graduate School, Daejeon University, 62 Daehak-ro, Dong-gu, Daejeon, 34520 Republic of Korea; 2 Department of Physical Therapy, College of Health and Medical Science, Daejeon University, 62 Daehak-ro, Dong-gu, Daejeon, 34520 Republic of Korea

**Keywords:** Back extensor fatigue, Electromyography, Gluteus maximus, Onset time, Isometric exercise, Trunk flexion ROM

## Abstract

**Background:**

Sacroiliac joints (SIJs) transmitted trunk load to lower extremities through the lumbopelvis. External compression devices across the SIJs could provide stability to the SIJs. A previous study established that using a device known as Active Therapeutic Movement version 2 (ATM^®^2) has been developed to improve pain and joint range of motion (ROM) in patients with LBP. However, no study has examined the physiological change in the muscle through ATM®2-based exercise thus far. This study aimed to determine the immediate effects of ATM^®^2 exercise on the contraction timing, back extension endurance, muscle fatigue, and trunk ROM of lumbar and lower limb muscles in healthy subjects.

**Methods:**

Thirty-six healthy subjects (mean age = 23.16 ± 2.3) volunteered to participate in this study. Subjects were instructed to perform ROM test using sit and reach test, back extensor endurance test using Biering-Sorensen test, erector spinae (ES), lumbar multifidus (LM) fatigue and onset time of Gluteus maximus (GM) in prone hip extension using electromyography before and after trunk flexion and extension isometric exercises.

**Results:**

The ROM in trunk flexion showed a significant increase of 7.9% after exercise compared to that before exercise (p < 0.05). Relative GM contraction onset timing significantly decreased after exercise (p < 0.05). The result of the Sorensen test after exercise showed a trend of increase in duration time. Muscle fatigue in the LM, however, showed a significant increase (p < 0.05), whereas muscle fatigue in the ES was reduced without statistical significance.

**Conclusions:**

The results base on this study showed that exercise-based on ATM®2 is an effective exercise protocol with an effect on the biomechanics of healthy subjects.

*Clinical trial registration numbers* KCT0006728. Clinical trial registration date: 09/11/2021.

## Introduction

From a three-dimensional perspective, the lumbopelvis is the center of the human musculoskeletal system. Trunk load is transmitted to the lower extremities via the sacroiliac joints (SIJs) through the lumbopelvis [1]. The surface orientation of the SIJs is consistent with the transfer direction of the trunk load, which can induce a high shear force between the sacrum and hip joint [2]. SIJs have a special anatomical structure and form a self-bracing mechanism to withstand external stimuli and loads caused by interaction with surrounding muscles and ligaments [3]. SIJs have a structurally flat shape and are surrounded by a strong ligament system that provides stability during vertical axial loading. However, ligaments are susceptible to creep under constant loads, and, from a biomechanical point of view, an additional active muscular system to enhance the stability of SIJs would be beneficial for SIJ ligaments [4, 5]. Weakening and inhibition of active systems such as the muscles around the lumbopelvis can prevent the effective transmission of loads from the low back to the lower extremities. The imbalance of muscles may cause SIJ dysfunction, which may further lead to low back pain (LBP) or pelvic girdle pain [6, 7]. A pattern of daily life involving excessive work, incorrect posture, and lack of exercise may cause problems such as weakening of back muscles, lack of flexibility, and poor balance. These problems lead to instability of the lower back, which may develop into more severe chronic low back pain [8, 9].

Core stability involves controlling trunk muscles that support the spine and trunk in a manner that maintains functional stability. Good core stability positively impacts posture, balance, strength, and coordination, and protects the body from damage. Structurally, the core is referred to as the lumbo-pelvic-hip complex. This complex includes both passive and active structures that produce or limit movement in the lumbar, hip, and pelvic segments [10]. In theory, wearing an external compression device across the SIJs could provide stability to the SIJs and the pelvis by compressing the joint surfaces together or by securing the SIJs in a fixed position [1]. Pelvic compression belts are commonly used clinically to increase pelvic stability and reduce pain [11, 12], and a pelvic compression belt was introduced in a recent study as a method to secure the stability of pelvic joints by utilizing the forced closure of the pelvis [13]. The muscles around the hip joint transmit force from the lower extremities to the trunk and provide stability to the lumbopelvis [14]. Pelvic compression belts compress the hip and SIJs to increase stability, thereby increasing the functionality of trunk movement and reducing compensatory mechanisms in the lower back during movement. As a result, muscle contraction occurs efficiently and muscle strength increases [15]. Kim et al. [16] investigated the effects of wearing a pelvic compression belt during a Swiss ball exercise program. Wearing a pelvic compression belt during Swiss ball exercise significantly increased back strength and flexibility in healthy college students. In patients with chronic low back pain, Oh [17] reported that muscle activity of the ES muscles and the right GM was significantly decreased during prone hip extension while wearing a pelvic compression belt. This suggests that a pelvic compression belt may improve the stability of the SIJ in patients with SIJ pain.

Exercise therapy is the most widely applied method of conservation therapy, and the physical therapist generally prescribes the exercise intervention [18]. The most frequently used exercises for patients with LBP are the McKenzie exercise, with a focus on lumbar extension exercise, and the William exercise, mainly comprising the flexion exercise [19]. A device known as *active therapeutic movement* version 2 (ATM^®^2) (BackProject Corporation, San Jose, California) has been developed to improve pain and joint range of motion (ROM) in patients with LBP. The exercise intervention based on ATM^®^2 could reduce pain by fixing the hip and lower chest area with a belt. Before fixation active contraction along the direction (flexion or extension) that caused the pain, and then perform the maximum isometric contraction of the trunk exercise within the painless range. Consequently, exercise within the painless range that restores the accurate position of the joint is likely to change muscle activity patterns controlled by the central nervous system to reduce pain caused during exercise [20]. Recently, there have been reported the results of applying ATM^®^2 in the treatment of patients with LBP clinically. The level of pain reduced and the ROM increased after several minutes of exercise.

Previous studies regarding ATM^®^2 were conducted on patients with kyphoscoliosis or scoliosis [21, 22]. In a case study by Nejishima et al. [20], where ATM^®^2 was applied in an intervention, a significant improvement was reported when the pain level was measured at 4 and 8 weeks compared to that before intervention. However, limited and poor-quality evidence of the study that has examined the physiological change in the muscle through ATM^®^2-based exercise thus far. Although various studies have evaluated the effects of additional external pressure on the pelvis, no study has evaluated the effects of a compression belt around the thoracic simultaneously during functional tasks. Thus, this study aimed to determine the immediate effects of ATM^®^2-based exercise on the contraction onset timing of GM, back extension endurance, back extensor muscle fatigue, and trunk ROM of lumbar in healthy subjects.

## Methods

### Subjects

The sample size was estimated by the objectives of this study using the Cohen’s D equation in the G*Power 3.1.9.2 program (University of Kiel, Kiel, Germany). We conducted a preliminary study on 10 subjects to maintain the testing power regarding the effects of ATM^®^2-based exercise, with the change in trunk flexibility before and after exercise as the primary indicator. Based on the data collected, the effect size was estimated at 0.65, and by setting the level of significance at 0.05 and the testing power at 0.95, the sample size was estimated to be n = 28. Considering a 20% drop-out rate, a minimum of 36 individuals were recruited.

The principal investigator verbally explained the study purpose and procedures to all participants before the experiments. All subjects adequately understood and voluntarily agreed to participate in the study. The inclusion criteria were as follows: (1) an individual without pain in the waist or lower limbs; (2) an individual capable of lumbar extension on muscle endurance measurement without pain or discomfort; (3) an individual with no adverse reaction on maximum tension of the belt during long hours of ATM^®^2; and (4) an individual who understands the study purpose and voluntarily agreed to participate. The exclusion criteria were as follows: (1) an individual diagnosed with an orthopedic, neurological, cardiorespiratory, or mental disorder within the past 3 months; (2) an individual who sustained a wound in the waist or lower limb area within the past 6 months; and (3) an individual who requested discontinuation due to pain or discomfort during the experiments.

### Experimental procedures

**Fig. 1:**
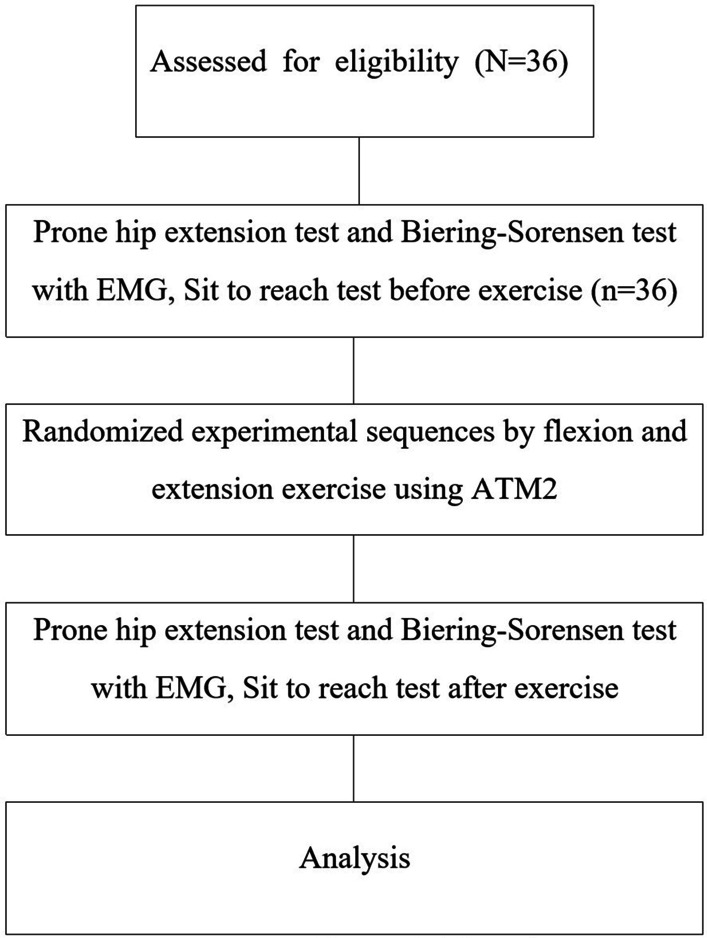
Flow chart

All subjects were requested to complete a questionnaire regarding the general characteristics and items related to the inclusion and exclusion criteria before the experiments. The selected subjects were then provided with explanations regarding the motions to be performed in the experiments, each of which were to be practiced at least three times for an accurate understanding of the methods involved in each exercise, before the experiments. To measure the trunk ROM, the subjects performed sit and reach (SR) exercise, and the sitting trunk flexion tester was used. To measure the SEMG, SEMG electrodes were attached to the ES, lumbar multifidus muscle (LM), GM, and HM on the dominant side. The Biering-Sorensen test was used to measure lumbar extension endurance; however, to measure the GM and HM contraction onset timing, the subjects performed PHE. Isometric extension and flexion exercises were performed using ATM^®^2. To set the performing order of isometric extension and flexion exercise for subjects, a randomizer (Research randomizer; http://www.randomizer.org/) was used before the experiments (Fig. 1). All tests were performed before and after exercise to determine the immediate effects of each exercise. The experimental protocol was established, according to the ethical guidelines of the Helsinki Declaration and was approved by the Human Ethics Committee of Institutional Review Board (IRB) of Daejeon University (IRB No. 1040647-202110-HR-011-03) and subsequently registered in the Clinical Research Information Service (CRIS; KCT0006728). Written informed consent was obtained from individual or guardian participants.

### ATM^®^2-based exercise

**Fig. 2:**
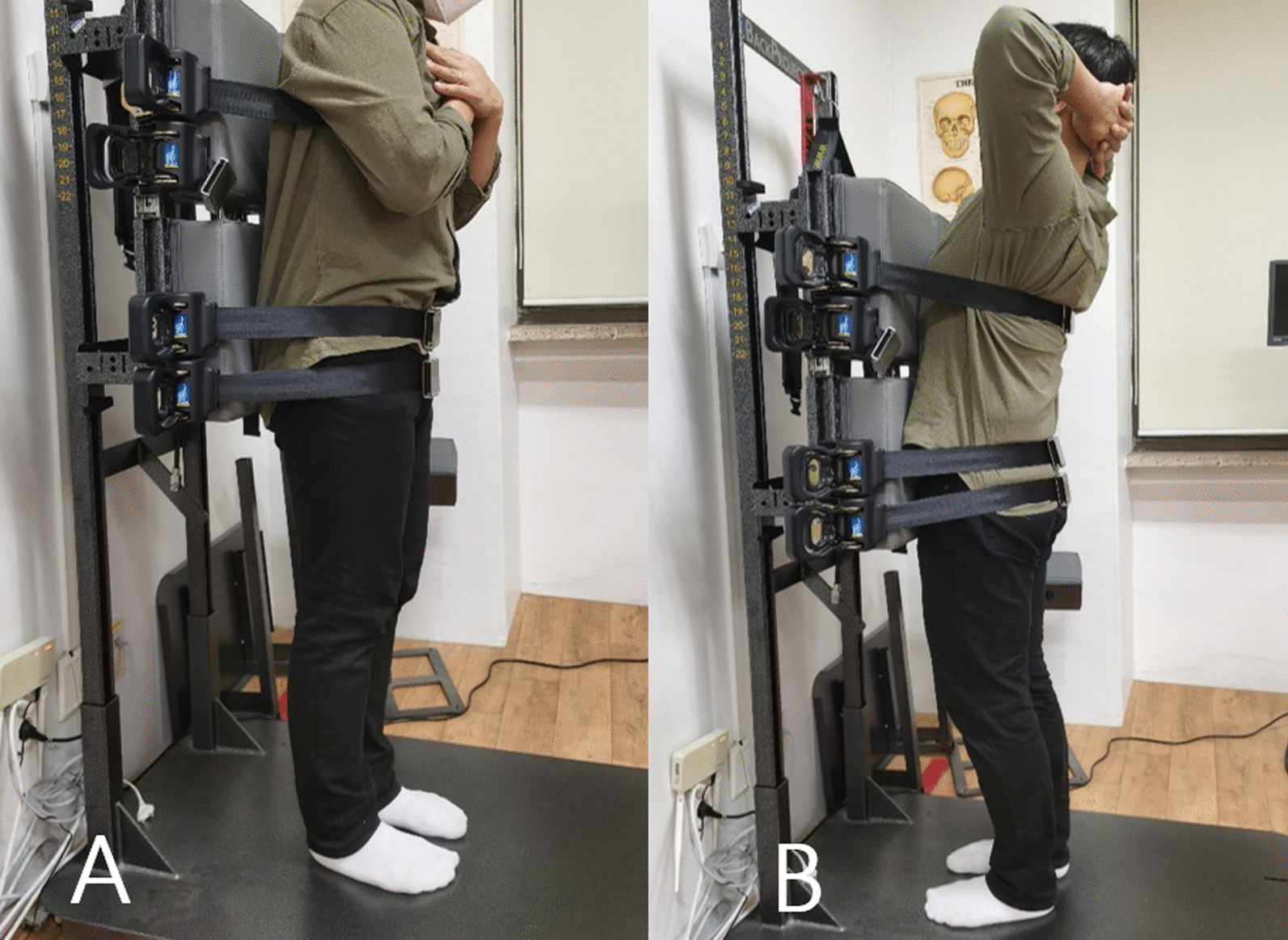
Isometric flexion and extension exercise using ATM^®^2. **A**: isometric flexion exercise; **B**: isometric extension exercise

For ATM^®^2-based exercise, subjects were instructed to perform trunk isometric flexion exercise or extension exercise in the standing posture (Fig. 2). The exercise direction was set to the one that elicited stronger pain at maximum flexion or maximum extension in the initial assessment [23]. As this study included healthy subjects, both flexion and extension exercises were performed. Each subject wore two belts around the hip area and one belt around the chest area for fixation. The hip belt had two parts—upper and lower; the upper part was placed around the anterior superior iliac spine (ASIS), while the lower part was placed around the greater trochanter. The chest belt was placed on the lower side of the chest. Belt compression was at a level that did not induce pain, and the maximum strength was controlled within a range that allowed stable breathing. Once the belts were in place, the subject was guided to perform an extension exercise with the maximum isometric contraction in the trunk extension direction with hands held behind the head. For the flexion exercise, the maximum isometric contraction was performed in the flexion direction as the subject held their hands before the chest. In a single exercise, a 3-s isometric contraction was repeated 10 times, and a 2-s break was allowed between each contraction task.

### Lumbar extension endurance

The Biering-Sorensen test, developed by Biering-Sorensen in 1984, was used to assess back extensor muscle endurance [24]. For the initial posture, the subject was guided to lay on the treatment table with the ASIS positioned at the edge of the table. The pelvis and legs were strapped in position using three belts in the areas close to the greater trochanter, knees, and ankles. Before the test, the subject was allowed to rest the torso on a chair. The measurements were taken for as long as the subject could maintain the posture of the extension of the upper trunk away from the low chair and place the arms across the chest. The subject should maintain the posture in the neutral trunk alignment. The rater measured the time that the subject could maintain and remained beside the subject to assist at the end of the test and during the break. The test was terminated when the subject could not maintain a posture of deviations greater than 10° in the sagittal plane or when the duration time was 240 s.

### Muscle fatigue

To collect data on muscle activity, the SEMG device (TeleMyo 2400 T, Noraxon, USA) was used to measure muscle activity and muscle contraction onset timing. A program with the designated software was used to analyze muscle activity of the GM, ES, and HM at the hip joint as well as the muscle contraction onset timing. The bandpass filter, filtration filter, and rate of sample extraction were set at 20–450 Hz, 60 Hz, and 1024 Hz, respectively. All collected data regarding muscle activity were analyzed using the root mean square (RMS) with a 50 ms moving window. Before electrode attachment, the target areas were shaved to minimize skin resistance and cleaned using an alcohol swab. A disposable Ag/AgCl surface electrode was attached to each target area.

Muscle fatigue in the lumbar region was measured through EMG during endurance exercise. The measured areas were the LM and ES. For the LM, the electrode was attached to an area 2 cm laterally away from the dominate side between L4-L5 spinous processes. For the ES, the electrode was attached to an area 3 cm laterally away from dominating side of the L3 spinous processes [25, 26]. EMG signals collected during the endurance test were analyzed based on the power spectrum through the fast Fourier transform. The overall gradient was estimated to obtain the resulting frequency data.

### Muscle contraction onset timing

To measure the muscle contraction onset timing, data on the HM and GM on the dominant side of PHE were collected. The HM electrode was attached to an area in the middle, between the center of the knee, elbow, and femoral region [27]. The GM electrode was attached to an area in the middle, between the greater trochanter and the sacrum, at 2 cm intervals on a diagonal line [28]. The hip joint extension was applied to the dominant leg. The subject was guided to lay in the prone position with the ankles aligned with the edges of the table in a neutral state. The arms were kept down in a comfortable position, while the back of the hand was kept upward in an anatomical posture. During hip joint extension, care was taken to prevent the legs from being adduction or abduction. All subjects were guided to ensure the knees are extended when performing hip joint extension. All measurements were repeated three times, and between each measurement, a 30-s break was given. The mean of the measurements was considered.

Muscle contraction onset timing was analyzed using MyoReaseach Master Edition 1.06 XP software (Noraxon, Scottsdale, AZ, USA). The data based on muscle activity analysis were RMS treated, and muscle contraction onset timing was analyzed using the designated program. Before PHE performance, the standard deviation (SD) for the mean of the EMG signal onset and the most stable 100-ms period were analyzed. The resulting values were applied in setting the relative muscle contraction onset timing as the first point of two consistent SD values above 25 ms. The GM contraction onset timing was set as the relative timing in the analysis. The HM contraction onset timing was set as the reference (0), the time from HM contraction up to GM contraction was set as the negative (–), and the time of in-advance GM contraction before HM was set as the positive (+) when measuring the GM contraction onset timing.

### Trunk flexion ROM

The SR test was used to assess the trunk flexion ROM. The testing tool was installed as the subject sat on a flat surface in preparation for the reach motion. The subject was guided to take off the shoes and sit with both legs outstretched and both heels tightly touching the testing tool. The subject was guided to place hands overlapped on the testing tool for the reach, with both arms outstretched. At the signal from the rater, the subject breathed out and slowly bent the upper body, while pushing the scale with fingertips as far as they can go. A slow and steady motion was maintained to prevent the gauge of the tester from being pushed by bouncing, holding the end position for approximately 2 s. If the subject displayed an inadequate understanding of the motion, the rater demonstrated the motion with the hands to induce the accurate motion. Any forced movements, such as excess push on the back or pull on the arms were prevented, and care was taken to prevent bending of the knees. Measurements were taken after an adequate level of practice. The values were recorded in centimeters for the maximum distance on the scale pushed by the subject as the upper body bent forward. The mean of duplicate measurements was considered for the final analysis.

### Data analysis

Statistical analysis of all collected data was performed using the SPSS version 20.0 for Windows (SPSS Inc., Chicago, IL, USA). Descriptive statistics were applied to analyze the general characteristics of the subjects; the mean and standard deviation were calculated for all measured variables. The Shapiro–Wilk test was used to test the normality of the measured variables. The paired t-test was used to compare the changes in the trunk extension endurance, back extensor muscle fatigue, trunk ROM, and GM, HM contraction on PHE before and after exercise. The level of significance was set at α = 0.05.

## Results

### General characteristics of subjects

Thirty-six subjects (22 men and 14 women) participated in this study, and the general characteristics, including the mean age, height, weight, and body mass index, are presented in Table 1. Across all measured variables, no sex differences were found; therefore, the data were analyzed without discrimination.

**Table 1:** General characteristics of subjects (n = 36)

Variables	Mean ± standard deviation
Gender (male/female)	22/14^a^
Age (year)	23.16 ± 2.30
Height (cm)	169.67 ± 9.89
Weight (kg)	67.60 ± 14.02
BMI (kg/m^2^)	23.33 ± 3.37

^a^Numbers, BMI; body mass index

### Comparison of trunk flexion ROM before and after isometric exercise using ATM^®^2

The ROM in trunk flexion was compared before and after isometric flexion and extension exercise using ATM^®^2 (Table 2). The trunk ROM showed a significant increase of 7.9% after exercise compared to that before exercise (p < 0.05).

**Table 2:** Comparison of range of motion in trunk flexion before and after isometric exercise using ATM2 (n = 36)

Pre	Post	t	*P-value*	Cohen’s d
6.03 ± 13.45	9.18 ± 12.61	− 7.601	< 0.001^*^	0.24

Values are presented as mean ± standard deviation (cm),*p < 0.05

### Comparison of trunk muscle endurance before and after isometric exercise using ATM^®^2

Trunk muscle endurance was compared before and after isometric flexion and extension exercise using ATM^®^2 (Table 3). The result of the Biering-Sorensen test after exercise showed a trend of increase in duration time, and muscle fatigue in the ES was reduced. However, muscle fatigue showed a significant increase (p < 0.05).

**Table 3:** Comparison of duration time and fatigue of back muscle activation before and after isometric exercise using ATM2 (n = 36)

	Pre	Post	T	*P-value*	Cohen’s d
Duration time (s)	75.62 ± 35.86	81.69 ± 31.07	− 1.094	0.281	0.18
Fatigue slope					
ES	− 1.39 ± 1.57	− 1.25 ± 1.00	− 0.562	0.578	0.11
MF	− 1.18 ± 1.13	− 1.63 ± 1.45	2.157	0.038^*^	0.35

Values are presented as mean ± standard deviation, ES; erector spinae, MF; multifidus, *p < 0.05

### Comparison of lower limb muscle contraction onset timing before and after isometric exercise using ATM^®^2

Table 4 presents the results of the comparison of the muscle contraction onset timing for trunk GM before and after isometric flexion and extension exercise using ATM^®^2. The results showed that the relative GM contraction onset timing significantly faster after exercise (p < 0.05). Hence, there was a significant faster in the interval between the GM and HM contraction onset timings after exercise.

**Table 4:** Comparison of the onset time of gluteus maximus activation before and after isometric exercise using ATM2 (N = 36)

Onset	Pre	Post	t	*p-value*	Cohen’s d
Time (s)	− 0.38 ± 0.37	− 0.28 ± 0.30	− 2.216	0.033*	0.3

Values are presented as mean ± standard deviation, *p < 0.05

## Discussion

This study was conducted to determine the immediate effects of isometric flexion and extension exercise with external compression through ATM^®^2 on lumbar extension endurance, back extensor muscle fatigue, lower limb muscle contraction onset timing, and trunk flexion ROM in healthy subjects. The results showed a significant increase in trunk flexion ROM after exercise and a significant decrease in the relative GM contraction onset timing. For lumbar extension endurance, the Biering-Sorensen test showed a trend of increase in the duration time on lumbar extension and muscle fatigue in the ES showed a trend of decrease after exercise. Muscle fatigue in the LM, however, significantly increased.

The ATM^®^2 device has four belts for the safe fixation of the user’s body onto a vertical treatment table. The belt strength can be controlled for different exercises at various angles. The patient is placed in an upright weight-bearing position that immediately and safely allows them to do fully resisted exercises in the direction and range of the previously impaired movement. The Central Nervous System governs the dynamic movement stability components, namely the neural, passive, active [29], and emotional components [30]. These components work throughout the anatomical structures of the musculoskeletal system. Normal dynamic stability provides the healthy body with the ability to perform normal, good quality, low-energy/high efficient movements. The passive holding is an essential setup for the ATM^®^2 because it will ensure that the superimposed active movement will alter the CNS activation strategy from pathological to normal. We believe that perhaps due to improved CNS governed dynamic stabilization of the specific movement. Increasing efficiency and usage of the core stabilization muscles cause immediate relaxation of global movement muscles which results in an immediate increase in ROM. Lewis et al. [22] investigated the effects of the exercise intervention using a program with ATM^®^2 and home exercise for 4 weeks in 43 patients diagnosed with scoliosis. The results showed an increase in the spinal ROM after the intervention, with improvements in pelvic alignment, pain, and disability. Patients with LBP have reduced lumbar ROM and proprioception with slower motion than healthy individuals [31]. Thus, therapists have used various treatment methods to increase lumbar flexibility. Mazloum et al. [32] reported a significant increase in the trunk flexion ROM through training based on pilates and extension exercise that significantly reduced pain in patients with LBP. In clinical practice, the use of ATM^®^2 in patients with LBP requires the belt tension strength to be set based on the limited ROM that pain provocation when performing trunk flexion or extension. As this study targeted healthy adult individuals, the belt tension strength during exercise was set to a maximum level without causing discomfort. Ito and Gamada [33] evaluated the trunk ROM using the finger-to-floor distance of healthy adult men between the group who performed ATM^®^2-based exercise and the control group after intervention. The results showed that the ROM significantly increased in the exercise group than in the control group. Nejishima et al. [20] also examined the effects of ATM^®^2-based exercise in 14 patients with LBP and reported that the level of pain and LBP disability (Roland-Morris Disability Questionnaire) had significantly decreased after exercise during the tests at week 4 or 8. Likewise, in this study, the SR test after exercise showed a significant increase in the trunk flexion ROM. The increase in trunk ROM through exercise suggested a potential intervention effect of the exercise on patients with LBP.

Lumbopelvis stability is considered a critical factor in the prevention and treatment of injury based on the potential contribution to recovery from injury and subsequent improvement [34]. For humans to maintain a straight posture, it is necessary to produce a force against gravity. The weights of the upper limbs, trunk, and head are conveyed to the pelvis via the spine. The pelvis connects the spine and muscles of the trunk to the lower extremities. The pelvis allows a person to tilt and rotate forward and backward as well as support the weight of the body. However, abnormal pelvic tilt may shift the body's center of gravity, leading to gradual weakening of the neuromuscular system [10]. Trunk muscles participate in the activities of the trunk and limbs, acting as synergistic or agonistic muscles for voluntary movements. Additionally, trunk muscles automatically respond to unexpected movements of the trunk and extremities and are involved in proactive posture control [35–37]. Since the trunk muscles play a role in maintaining postures in daily life, the strength and endurance of the trunk muscles must be maintained. One exercise for pelvic training is a trunk stabilization exercise that simultaneously activates the abdominal muscles and the local muscles of the spine for coordinated movements [38]. Many previous studies focused on lumbopelvis stabilization and suggested methods to strengthen core stability, such as trunk stabilization exercises, training using biofeedback mechanisms, and self-training from a cognitive perspective [39]. However, it is challenging for an individual to become aware of and control core-stabilizing muscle groups. Several methods have been suggested in previous studies for trunk stabilization [40, 41], including an abdominal draw-in maneuver, which is an exercise for lumbar stabilization. According to a previous study, this exercise shows the best stabilization effects in transverse abdominal muscle and multifidus muscle [42]. Activation of the abdominal muscles is essential in stabilizing the pelvis against the pulling forces of the hip muscles. When the pelvis is stable, forces acting on the trunk are efficiently transmitted to the hip and lower extremities [42]. Also, to maintain stability in the pelvis, the force of compression should be increased [43]. Most treatments for patients with LBP include trunk muscle strengthening training, and recently, core stabilization exercise has frequently been applied [44]. In addition, an external compression device may be used to provide stability. Arumugam et al. [45] reported that the external compression on the pelvis enhanced the form closure of the sacroiliac joint as well as the forced closure and exercise control through reduced compensation on lumbar stability muscles. In another previous study, the use of a pelvic compression belt affected the thickness of the LM and ES have been proved through ultrasound. Its potential use as an effective assistive tool in muscle strengthening exercise [46]. Previous studies have investigated the effects of pelvic compression belts on the body during various treatments. Shin et al. [47] reported that hip abduction muscle strength was significantly greater in the group that wore pelvic compression belts than in the control group after two weeks of manual treatment in total knee arthroplasty patients. The ATM^®^2 device in this study may provide a pelvic compression effect during the isometric exercise. The application of the device to patients with LBP is anticipated to produce more effective therapeutic effects than the common muscle strengthening exercise.

The lumbar region has an important dynamic function of supporting the upper trunk while conveying the compression and shearing forces that arise during daily activities to the lower limbs [48]. This provides spinal stability that is maintained based on the passive support from the bone and ligament structure, active support from the muscles, and interactions among the control systems via the central nervous system [29]. Previous studies reported that, in patients with LBP, the limb or trunk exercise led to a different type of exercise control from that in healthy subjects [13, 49, 50]. In patients with LBP, compared with healthy subjects, the trunk muscle activity and the order of contraction onset timing changed [13]. Causes spinal movement involved multi-segment joints, the limitation of a single segment to increase the movement of another segment in compensation, and movement of the limbs is related to the movement of the spine [51]. Janda and Jull [52] reported that on PHE, the normal order of muscle activity is the contraction of the GM preceding the contraction of the HM and that the change in this order would induce excess compensatory lumbar extension, thus resulting in weakening of the GM. In contrast, Pierce and Lee [53] reported that on hip joint extension, the order of muscle contraction was consistently initiated by the HM before extension. Kwon and Koh [51] also reported that on PHE or hip joint extension in a standing posture, the HM began contraction first before the GM. Similarly, in this study, GM contraction preceded HM contraction before and after exercise in two subjects, while all other subjects showed the HM contraction first. Kwon and Koh [51] reported that on PHE, GM contraction was significantly delayed compared to HM contraction in the LBP group; the delay was by 0.03 s in the control group and 0.18 s in the LBP group. The measurements for PHE in this study showed that the mean delay in GM compare to HM was 0.38 s before exercise and 0.28 s after exercise, with a significant difference in muscle contraction onset timing before and after exercise. For PHE, it is easy for the HM to be overused when it becomes the dominant extensor as the GM is unable to function adequately, which can ultimately lead to LBP [54]. In a study by Hungerford et al. [55], patients with sacroiliac joint pain were guided to stand on one leg and perform the hip flexion on the other leg and as the biceps muscle contraction occurred first, a delay in the GM contraction was observed. Thus, it is presumed that the adjustment of the order of muscle activity has a critical role in the prevention and treatment of LBP.

The spine itself is an unstable structure with appropriate support from the surrounding muscles and tissues for compensation [56]. Regarding muscle endurance in the lumbar region, muscle fatigue is a frequently detected problem in patients with LBP and a risk factor for LBP [57–59]. Thus, the state of the trunk can be accurately determined through the assessment of lumbar muscle function and fatigue to provide suitable treatments. The Biering-Sorensen test in this study was used to assess the muscle endurance around the lumbar region based on the duration time and EMG. In a study by Jung et al. [60], the group that received the 4-week manual therapy and the group that received exercise therapy showed reduced pain after the intervention, while trunk stability and duration time in the Biering-Sorensen test increased. This study hypothesized that the duration time in the Biering-Sorensen test would increase after exercise to decrease muscle fatigue due to test motions. The results of this study showed a trend of increase, despite the lack of significance, in the duration time after exercise. In addition, muscle fatigue in the ES decreased, although without statistical significance. However, contrary to the hypothesis, muscle fatigue in the LM increased after exercise. This was attributed to the difficulty in performing test motions repeatedly within a short period between two measurements, despite the adequate resting time. Furthermore, in the Biering-Sorensen test used in this study, the subjects were guided to maintain posture up to as high as 120 s. For some subjects who could go beyond 120 s, the experiment ended as they reached 120 s to prevent potential influence on the statistical results. In addition, this study aimed to analyze the immediate effects of exercise intervention, and performing a single exercise might have prevented the predicted effects from being reached. In the future, the effects of a long-term exercise intervention with a 2- or 4-week training program in terms of muscle endurance and muscle fatigue should be investigated. Moreover, previous studies show that surface electromyography cannot fully measure deep muscles such as multifidus muscle [61]. However, since multifidus muscle consists of both deep and surface segments [62], the activity of multifidus muscle has been measured using surface electromyography in many previous studies [63–65]. In this study, to avoid invasive procedures, the activity of multifidus muscle was measured using surface electromyography as previously described. In follow-up studies, it will be important to use methods such as needle electromyography for a more accurate assessment of muscle activity.

This study has several limitations. First, only the immediate effects of ATM^®^2-based isometric flexion and extension exercise were evaluated. Second, the subjects were not patients with LBP, but healthy individuals. Third, the results may have been influenced by subjective influencing factors during the procedures as they could not be completely excluded. In future studies, these limitations should be compensated to procure more scientifically valid clinical data.

## Conclusion

This study investigated the immediate effects of isometric flexion and extension exercise on the contraction onset timing, back extension endurance, muscle fatigue, and trunk ROM of the lumbar and lower limb areas in healthy subjects. The exercise was performed using ATM^®^2 with external compression on the hip area for fixation. The results showed a significant increase in the trunk ROM after exercise, while the relative contraction onset timing in the lower limb GM was significantly reduced. Furthermore, the muscle endurance test after exercise showed a trend of increase in the duration time with a decreasing trend in muscle fatigue in the ES.

In conclusion, isometric exercise based on ATM®2 is an effective exercise protocol with an effect on the biomechanics of healthy subjects. This study provides a good starting point for discussion and further research on which long-term effects of the exercise. Future research should have to confirm whether this exercise might be applied in clinical practice for patients with LBP.

## Data Availability

The datasets used and/or analyzed during the current study are available from the corresponding author on reasonable request.

## Abbreviations

ASIS: Anterior superior iliac spine
ATM^®^2: Active therapeutic movement version 2
CRIS: Clinical research information service
ES: Erector spinae
GM: Gluteus maximus
HM: Hamstring
IRB: Institutional review board
LBP: Low back pain
LM: Lumber multifidus
PHE: Prone hip extension
RMS: Root means square
ROM: Range of motion
SD: Standard deviation
SEMG: Surface electromyography
SR: Sit and reach
